# Development of the evidence-based guidelines for home birth in Switzerland: Results from a Delphi process

**DOI:** 10.18332/ejm/220976

**Published:** 2026-08-31

**Authors:** Adrien Bruno, Piroska Zsindely, Rafaela Joos, Myrim Barbara Rösch, Carole Lüscher, Charlotte Farin-Moënnat, Claire Ajoubair, Anne Steiner

**Affiliations:** 1 School of Health Sciences, University of Applied Sciences and Arts Western Switzerland, Lausanne, Switzerland; 2 School of Health Sciences, Zurich University of Applied Sciences, Winterthur, Switzerland; 3 Ayor – ganzheitliche Hebammenpraxis, Rumlikon, Switzerland; 4 Hebammenpraxis Myrim and Team GmbH, Bern, Switzerland; 5 Hebammenpraxis 9punkt9 GmbH, Bern, Switzerland; 6 Birthing center ‘Graines de Vie’, Corsier-sur-Vevey, Switzerland; 7 Birthing center ‘La Grange Rouge’, Grens, Switzerland; 8 Quality and Innovation, Swiss Federation of Midwives, Olten, Switzerland

**Keywords:** home childbirth, midwifery, practice guidelines as a topic, risk assessment, birth setting

## Abstract

In Switzerland, the right to a home birth is enshrined in law, and the associated costs are reimbursed by the national health insurance system. However, the conditions under which such births should occur remain unregulated at the national level, with no shared criteria for determining eligibility across the country. The aim of this project is to develop national guidelines to define criteria under which pregnant women would be ineligible for a planned home birth.

A four-phase process was conducted, combining scientific evidence and expert consensus. It began with a systematic review of international home birth guidelines, conducted across five guideline specific databases and registries, complemented by a manual search. Subsequently, the identified criteria were submitted to expert classification by experienced Swiss home birth midwives through an online survey and three regional Delphi inspired workshops. Finally, the draft guideline was circulated to all members of the Swiss Federation of Midwives (SFM) for national consultation.

The systematic search identified 262 records, of which 249 were screened and 42 assessed in full text. Nine guidelines met the inclusion criteria, from which 97 unique ineligibility criteria were extracted. Thirty Swiss clinical midwives participated in the classification phase, resulting in a four category structure distinguishing absolute ineligibility from situations requiring individual assessment. The national consultation generated 433 responses, leading to refinements and clarification of several criteria. The final guidelines comprise 97 criteria, supported by structured decision making pathways. These criteria include both medical conditions (e.g. unmanaged hypertensive disorders) and contextual factors requiring individual assessment (e.g. history of a previous baby >4500 g).

This policy initiative has led to the first nationally endorsed home birth guidelines in Switzerland. Built on scientific evidence and clinical consensus, these guidelines provide a harmonized framework to support safety and professional autonomy in midwifery practice.

## INTRODUCTION

The midwifery profession is key to ensuring maternal and infant health, both in Switzerland and internationally. Numerous studies have highlighted that midwife-led and continuity-of-care models are associated with fewer medical interventions (e.g. cesarean section, episiotomy), enhanced maternal and neonatal outcomes, higher family satisfaction, and cost-effectiveness^1-4^. Despite these proven benefits, ensuring consistent and safe midwife-led care – particularly in out-of-hospital settings – requires clear and evidence-based professional frameworks.

In Switzerland, the responsibilities of midwives in pregnancy and birth care vary significantly depending on the chosen place of birth^5^. Pregnant women can choose to give birth in a hospital, a birth center, or at home, and all of these options are covered by the national health insurance system^6^. While hospital births are strictly governed by institutional protocols, birth centers are also commonly considered recognized healthcare facilities^7^. In 2023, 1.2% of births in Switzerland took place in the family home^8^.

Although the right to a home birth is enshrined in Swiss law and the associated costs are reimbursed by the national health insurance system^9^, such births are not currently supported by any official national recommendations. The midwives who provide this care practice independently and in diverse professional contexts, making it difficult to harmonize practices. This lack of a shared reference framework poses challenges in terms of safety, quality of care, and professional recognition. The SFM therefore took action to strengthen the quality of care in the field of home birth^10^.

The SFM is the sole national professional association representing midwives in Switzerland^11^. As of 2024, it brings together over 3500 members across the different linguistic regions of the country, including 2684 in the German-speaking part, 751 in the French-speaking part, and 88 in the Italian-speaking part^12^. The Federation plays a key role in promoting high-quality midwifery care^13,14^, supporting continuing professional education^15^, and influencing health policies related to maternal and newborn care^16,17^. The SFM also oversees quality monitoring and maintains statistical records on midwifery practice and birth settings throughout the country^8^.

In response to the diversity of individual practices and the absence of shared selection criteria to determine which situations are appropriate for home birth, the aim of this project is to develop a national list of ineligibility criteria for home birth, based on scientific evidence.

By focusing on ineligibility criteria, we can avoid unnecessarily restricting access to home birth for women who are otherwise good candidates for it, promoting a more individualized approach to care. To ensure scientific rigor in the process of developing such criteria, the SFM appointed a working group composed of academic and clinical midwifery experts. This article outlines the development process, methodological framework, and rationale behind the creation of these national guidelines.

## COMMENTARY

The development of Swiss national guidelines for selecting pregnancies for which home birth would be suitable was conducted between July 2024 and May 2025. The process unfolded in four main phases.

### Phase 1: Literature review

The initial phase consisted of a systematic review of existing international guidelines on eligibility for home birth, conducted by AB, PZ and AS, between July and September 2024. The following databases and repositories were searched, combining subject headings and free-text terms to identify relevant guidelines: Guideline Central, Guideline International Network, la Haute Autorité de Santé, Joanna Briggs Institute EBP Ovid, and Trip medical database (Supplementary file). Keywords consisted of ‘home birth’ and ‘guideline’ and their synonyms. Manual searches were conducted for other professional societies and grey literature, including position papers and consensus statements.

A total of 262 references were imported for screening. After duplicates were removed, 249 records were screened based on their titles and abstracts. Two reviewers independently assessed each record, and, in cases of disagreement, discussions were held amongst the three reviewers until a consensus was reached. Of these 249 articles, 42 met the following predefined criteria. These criteria required that publications must explicitly focus on out-of-hospital or home birth, represented a set of international guidelines, were published in English, French, Italian, German, or Dutch, and provided evidence supporting their methodological validity.

After that, the full text of 42 sets of guidelines was assessed for relevance to our project. Of these, 33 were excluded, primarily because they were not relevant to home birth, lacked supporting evidence, or addressed system-level rather than individual eligibility criteria. During the full-text assessment, the evaluation method was the same as for the screening phase, except that four senior clinical midwives with expertise in home birth joined the team of reviewers. The full text of each set of guidelines was evaluated by one senior clinical midwife and one academic midwife.

**Figure 1:**
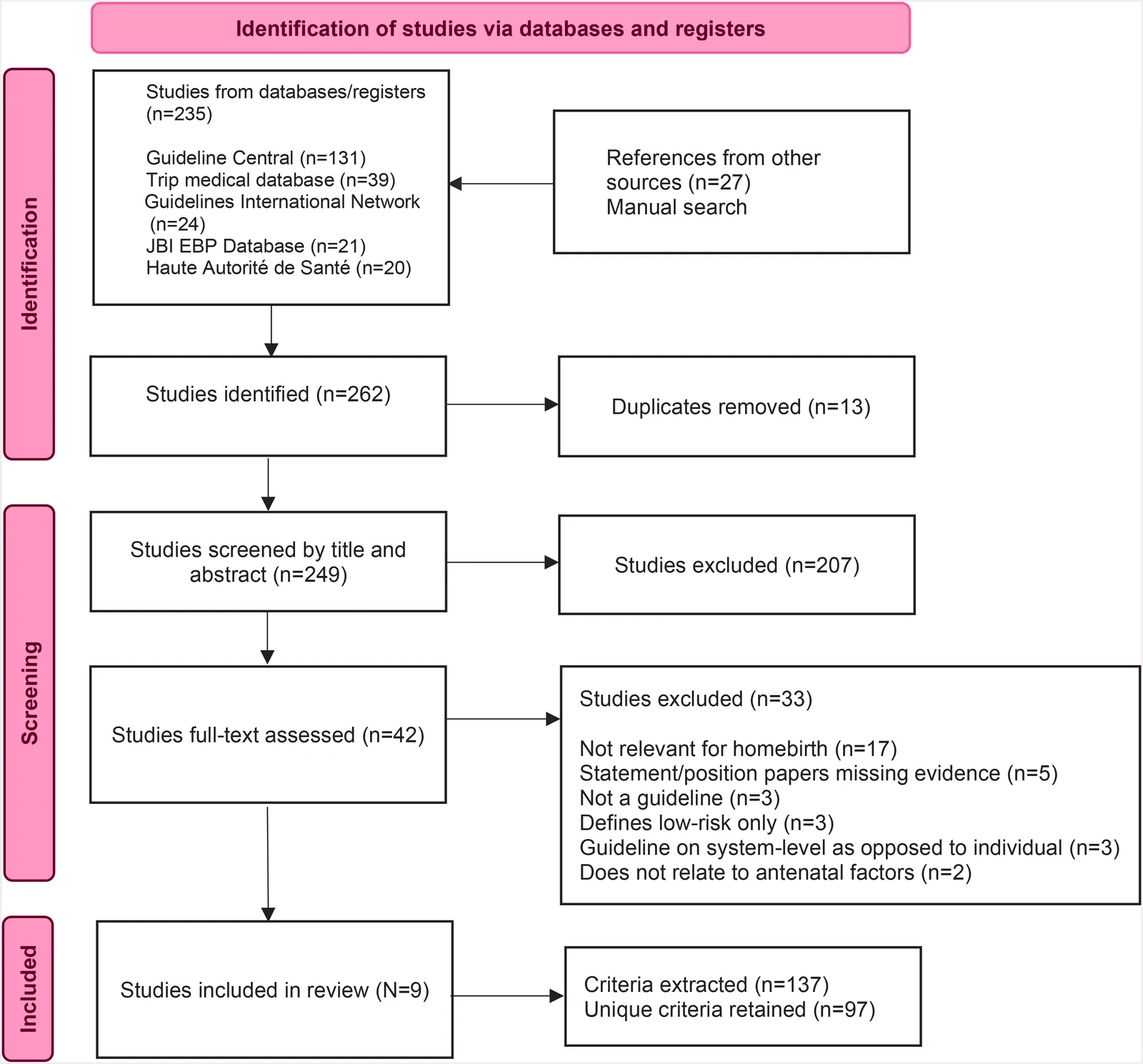
PRISMA flow diagram of the systematic literature review conducted across five guideline databases and manual sources between July and September 2024 to identify evidence-based home birth eligibility criteria

Ultimately, nine sets of guidelines^18-26^ met the inclusion criteria. From these, the team extracted 137 potential ineligibility criteria for home birth. After merging duplicates, a list of 97 unique criteria was established (Figure 1). The final list was subsequently translated by AB and PZ into French and German to be used in the next phase of the process.

### Phase 2: Expert classification

The second phase of the project was performed between October and December 2024, which involved the systematic classification of the 97 ineligibility criteria identified in the literature review. To ensure a broad and practice-oriented perspective, additional experienced clinical midwives were recruited through the SFM to participate in three workshops. To be eligible to participate, they had to have more than 5 years’ experience as midwives performing home births, and at least 40% of their clinical practice had to involve home birth care. The second-phase process was inspired by the Delphi method, a structured consensusbuilding technique in which experts independently assess predefined items across iterative rounds and receive anonymized feedback between rounds, allowing controlled interaction and progressive movement toward consensus^27^.

*Online questionnaire*

Prior to the in-person workshops, the 30 invited clinical midwives completed an online questionnaire designed to accelerate the process and ensure participants were familiar with the proposed criteria. They were asked to classify each criterion as either absolute (care in an obstetric unit would be expected) or relative (requiring further individualized assessment).

*Regional workshops*

Three regional workshops were then organized to facilitate in-depth discussion and collective decision-making about the home birth-related criteria, in Lausanne (6 participants), Bern (12 participants), and Winterthur (10 participants). During these workshops, each criterion was discussed and voted upon. When a criterion was considered relative, participants were asked to specify the type of assessment required to ensure safe decision-making.

*Consensus building*

At the conclusion of the workshops, most of the 97 criteria had been categorized by a majority of the participants as either absolute or relative. For criteria lacking a clear majority, the eight authors reviewed the results and sought consensus through discussion and by consulting evidence. While certain criteria had to be adapted to the Swiss context, none of the 97 ineligibility criteria identified in the literature was excluded.

This structured process ensured that the classification of criteria combined scientific evidence with the practical expertise of midwives specialized in home birth care, providing a balanced set of guidelines that is appropriate for the Swiss context.

### Phase 3: National consultation

The third phase, conducted in March 2025, involved a nationwide ballot of all members of the SFM. An online survey was distributed in French and German, along with a message explaining the rationale and importance of establishing unified national criteria for home birth. Respondents were invited to review the proposed list of criteria and were encouraged to indicate any disagreement, supported by relevant scientific evidence. By April 2025, 433 responses had been received.

### Phase 4: Finalization and structure of the guidelines

In the final phase, the working group reviewed the feedback received during the national ballot and finalized the guidelines. The comments submitted by SFM members mainly led to minor clarifications of wording, improved consistency across categories, and the addition of brief contextual explanations for a few criteria. No new evidencebased arguments emerged that warranted adding or removing criteria, but the consultation helped refine the formulation of the final guideline and ensured that the list reflected both professional consensus and practical usability.

Finally, the 97 criteria were classified into four categories following the National Institute for Health and Care Excellence guidelines, which served as the main reference framework for this study. The first category consists of medical conditions indicating increased risk and suggesting that the birth should occur in a hospital environment (Table 1), while the second comprises other factors indicating increased risk and suggesting that the birth should occur in a hospital environment (Table 2). The third category includes medical conditions indicating that individual assessment is needed when deciding on the place of birth (Table 3), and the fourth encompasses additional factors that indicate the need for individual assessment when deciding on the place of birth (Table 4). The relevant references are given in square brackets within each Table.

**Table 1:** Medical conditions indicating increased risk and suggesting planned birth in a hospital environment

*Area of disease*	*Medical condition*
**Cardiovascular**	• Hypertensive disorder – essential or gestational hypertension [18, 22]
**Hematological**	• Anemia – hemoglobin less than 85 g/L at onset of labor [19] • Immune thrombocytopenia purpura or other platelet disorder or platelet count below 100×109/L [19] • Von Willebrand's disease [19]
**Endocrine**	• Insulin dependent diabetes [18–20, 22]
**Infective**	• Hepatitis B or C – with abnormal liver function tests [19] • Tuberculosis under treatment [18, 19]
**Gastrointestinal**	• Liver disease associated with current abnormal liver function tests [19]
**Psychiatric**	• Severe mental health disorder requiring multiple psychotropic medications* [21] • Psychiatric disorder requiring current inpatient care [19]

* In all circumstances, the midwife must assess all medications used by the patient.

**Table 2:** Other factors indicating increased risk and suggesting planned birth in a hospital environment

*Factor*	*Additional information*
**Previous complications**	• Previous uterine rupture [19]
**Current pregnancy**	• Current placental abruption [18, 19] • Current pre-eclampsia [18, 19] • Current preterm labor (active labor before 37+0 weeks gestation) [18, 19, 22, 24] • Multiple birth [18, 19, 22, 24] • Breech or transverse presentation [18, 19, 23, 24] • Oligohydramnion with additional complicating factors [18] • Placenta previa [18, 19] • Alcohol dependence requiring assessment or treatment [18, 19] • Intrauterine infection [22] • Intrauterine growth restriction (IUGR) <5th percentile [18, 19, 23] * • Evidence of congenital fetal anomalies requiring immediate assessment and/or management by a neonatal specialist [18, 19, 22] • Pharmacological induction of labor [18, 19] • Rhesus isoimmunization [18]

* Deutsche Gesellschaft für Gynäkologie und Geburtshilfe & Deutsche Gesellschaft für Hebammenwissenschaft guideline (2020) mentions IUGR as risk but does not recommend a cutoff percentile. In the National Institute for Health and Care Excellence guideline (2023), the recommended cut off is the 3rd percentile.

**Table 3:** Medical conditions indicating individual assessment is needed when planning place of birth

*Area of disease*	*Medical condition*	*Recommended assessment*
**Cardiovascular**	• Confirmed cardiac disease [19, 22, 25, 26]	Assessment in collaboration with a specialist doctor
**Hematological**	• History of thromboembolic disorders [19] • Haemoglobinopathies, such as sickle-cell [19] • Sickle-cell trait [19, 22] • Thalassemia trait [19] • Atypical antibodies not putting the baby at risk of hemolytic disease [19]	Assessment in collaboration with a specialist doctor
	• Atypical antibodies which carry a risk of hemolytic disease of the newborn [19] • Bleeding disorder in the woman or unborn baby [19]	Assessment in collaboration with a specialist doctor (pediatrician)
**Endocrine**	• Hyperthyroidism [19, 25] • Unstable hypothyroidism such that a change in treatment is needed [19]	Assessment in collaboration with a specialist doctor
**Gynecological**	• Female genital mutilation [25]	Assessment in collaboration with a senior home birth midwife or quality circle
	• Fibroids (e.g. uterine myoma) [19] • Cone biopsy or large loop excision of the transformation zone [25] • Myomectomy [19] • Hysterotomy other than caesarean section [19]	Assessment in collaboration with an obstetrician-gynecologist
**Infections in current pregnancy**	• Genital herpes (initial infection or recurrence) [18, 19]	Assessment by the midwife
	• Human immunodeficiency viruses (HIV) carrier or infected with HIV [18, 19]	Assessment in collaboration with a specialist doctor
	• Chickenpox/shingles [19] • Rubella [19] • Syphilis [18] • Toxoplasmosis – women receiving treatment [19]	Assessment in collaboration with an obstetrician-gynecologist and pediatrician
**Immune**	• Non-specific connective tissue disorder [19] • Scleroderma [19] • Systemic lupus erythematosus [19]	Assessment in collaboration with a specialist doctor
**Skeletal**	• Spinal abnormalities [19] • Previous fractured pelvis [19]	Assessment in collaboration with a specialist doctor
**Gastrointestinal**	• Crohn's disease [19] • Liver disease without current abnormal liver function tests [19] • Hepatitis B or C – with normal liver function tests [19] • Ulcerative colitis [19]	Assessment in collaboration with a specialist doctor
**Respiratory**	• Cystic fibrosis [19] • Asthma requiring an increase in treatment or hospital treatment [19]	Assessment in collaboration with a specialist doctor
**Neurological**	• Epilepsy [19] • Myasthenia gravis [19] • Neurological deficits [19] • Previous cerebrovascular accident [19]	Assessment in collaboration with a specialist doctor
**Renal**	• Abnormal renal function [19] • Renal disease requiring supervision by a renal specialist [19]	Assessment in collaboration with a specialist doctor
**Mental health**	• Under current outpatient psychiatric care [19]	Assessment in collaboration with a specialist doctor

**Table 4:** Other factors which indicate individual assessment

*Factor*	*Additional information*	*Recommended assessment*
**Previous complications**	• Stillbirth or neonatal death with a known, non-recurrent cause [19] • Prior cesarean section [18, 19, 24] • Macrosomia – history of previous baby >4500 g [19] • Previous baby with jaundice requiring exchange transfusion [19]	Assessment by the midwife
	• Previous placental abruption – with good outcome [19] • Pre-eclampsia developing at term in last pregnancy [19] • Previous primary postpartum hemorrhage requiring additional procedures [18, 19] • Previous retained placenta needing manual removal in theatre [19] • Previous shoulder dystocia [18, 19] • Unexplained stillbirth and/or neonatal death, or previous death related to intrapartum difficulty [18, 19]	Assessment in collaboration with a senior home birth midwife or quality circle
	• Extensive vaginal, cervical or 4th degree perineal birth trauma in previous birth [19]^a^ • Previous placental abruption – with adverse outcome [19] • Eclampsia [19] • Pre-eclampsia/HELLP syndrome requiring preterm birth [19, 22]	Assessment in collaboration with an obstetrician-gynecologist
	• Previous baby with encephalopathy [19]	Assessment in collaboration with a specialist doctor
**Current pregnancy**	• Age >40 years at beginning of pregnancy [19] • Group b streptococcus positive where intrapartum intravenous antibiotics are recommended [19]	Assessment by the midwife
	• Anemia – hemoglobin 85 to 105 g/L at onset of labor [19] • Antepartum bleeding of unknown origin (single episode after 24 weeks of pregnancy) [19] • Grand multiparity – parity ≥5 [19, 25]^b^ • Macrosomia – clinical or ultrasound suspicion [19, 20] • Post term more than 41+6 weeks [18, 24, 25] • Suspected small for gestational age (SGA), <3rd percentile [19, 22, 23]^c^ • Recreational drug use^d^ [19]	Assessment in collaboration with a senior home birth midwife or quality circle
	• Body mass index ≥35 kg/m^2^ [19] • Recurrent antepartum hemorrhage [19] • Blood pressure ≥140 mmHG systolic or • ≥90 mmHG diastolic on two occasions [19] • Intrauterine death [19] • Intrauterine growth restriction (IUGR) ≥5th percentile [18, 23]^e^ • Ultrasound diagnosis of oligo- or poly-hydramnion [18, 19, 23]	Assessment in collaboration with an obstetrician-gynecologist
	• Abnormal fetal heart rate [19, 22] • Fetal abnormality [19, 22]	Assessment in collaboration with an obstetrician-gynecologist and pediatrician
	• Substance misuse during pregnancy [18, 19, 22]^f^ • Medical conditions that have required acute medical supervision during pregnancy [18]	Assessment in collaboration with a specialist doctor

a National Institute for Health and Care Excellence Guideline (2023) include 3rd degree tear.

b National Institute for Health and Care Excellence Guideline (2023) suggest grand multiparity of ≥4 to be at increased risk.

c Deutsche Gesellschaft für Gynäkologie und Geburtshilfe & Deutsche Gesellschaft für Hebammenwissenschaft guideline (2020) mentions SGA as risk but does not recommend a cutoff percentile.

d The term ‘recreational drug use’ refers to any type of consumption of legal or illegal psychoactive substances which occurs occasionally or regularly for recreational purposes. The term is therefore used in contrast to dependent drug use or substance misuse.

e Deutsche Gesellschaft für Gynäkologie und Geburtshilfe & Deutsche Gesellschaft für Hebammenwissenschaft guideline (2020) mentions IUGR as risk but does not recommend a cutoff percentile.

f Substance misuse, also known as drug abuse, is a patterned use of a drug in which the user consumes the substance in amounts or with methods which are harmful to themselves or others, and is a form of substance related disorder.

The recommendations for evaluating relative criteria (Tables 3 and Table 4) involved one of the following: assessment by the primary midwife; joint assessment with an experienced home birth midwife or within a quality circle (method of quality improvement in primary care^28^); or assessment in collaboration with a medical specialist.

In addition to refining the list of criteria, the team also drafted a preamble and a concluding section to support the appropriate use of the guidelines in clinical practice. The preamble outlines the rationale and scope of the document, emphasizing that planned home birth can be a safe and valid option when supported by adequate selection criteria and professional midwifery care. It should also be acknowledged that situations may arise where care is provided outside the defined guidance, particularly when this results from the woman’s informed decision, a complex issue that goes beyond the scope of this article.

The finalized guidelines, published in May 2025, are available in both French and German^29^ and are intended to serve as a national reference for the safe and consistent planning of home births in Switzerland.

### Strengths and limitations

This initiative represents a significant step forward in supporting the safe and harmonized practice of home birth in Switzerland. It resulted in the development of a national set of evidence-based guidelines specifically designed for planned home births. However, several limitations and considerations must be acknowledged.

One key issue concerns how out-of-hospital birth is defined. In the international literature, distinctions between midwifery-led units, birthing centers, and home birth are not always clearly defined. Although the set of Swiss guidelines established here was developed specifically for planned home births, some criteria may overlap with those relevant to broader out-of-hospital settings. This should be taken into consideration when interpreting and applying the recommendations in clinical practice.

Another important aspect is the dynamic nature of clinical care. As practices evolve and new evidence emerges, the guidelines must be adapted. To achieve this, the current list of ineligibility criteria will be regularly reviewed and updated to ensure it continues to reflect the best available evidence and actual clinical conditions in Switzerland.

### Implications

The success of these guidelines will ultimately depend on their effective implementation. To support this, the SFM, together with its cantonal sections, has proactively engaged with cantonal health departments to promote awareness and encourage institutional support. Dissemination efforts are also being directed towards midwives, healthcare providers, and educators involved in perinatal care. Finally, to ensure wider accessibility and usability across Switzerland, a translation into Italian is currently in progress.

## CONCLUSION

This article describes the structured, multi-phase process through which the SFM developed the first national guidelines defining ineligibility criteria for planned home birth. Grounded in international literature and refined through expert clinical input and national consultation, the final list of criteria provides consistent, evidence-informed guidelines to support safe and autonomous decision-making in midwifery-led care. The implementation of these guidelines, accompanied by periodic updates and broad dissemination, should contribute to strengthening the quality and coherence of home birth practices across Switzerland.

## Supplementary Material



## Data Availability

The data supporting this research are available from the authors on reasonable request.
