# Supplementary material for: Development of the evidence-based guidelines for home birth in Switzerland: Results from a Delphi process
Source: Eur J Midwifery. 2026 Aug 31;10:10.18332/ejm/220976. doi: 10.18332/ejm/220976 (PMC13539762; doi:10.18332/ejm/220976)
Supplement: Supplementary file 1 [file EJM-10-37-s1.pdf]

EUROPEAN JOURNAL
OF MIDWIFERY

Supplementary file

© 2026 Bruno A. et al.

DOI:
10.18332/ejm/220976

The content has been provided by the author(s) and has not been reviewed, verified, or endorsed by European Publishing. It may not have undergone peer review. The views, opinions, and recommendations expressed are solely those of the author(s) and do not necessarily reflect the position of European Publishing. European Publishing accepts no responsibility or liability for any consequences arising from the use of, or reliance on, this content.

Supplementary File 1. Methodological details of Phase 1: Systematic review of international home birth eligibility guidelines

Search strategy

A systematic search was conducted between July and September 2024 to identify international guidelines addressing eligibility for planned home birth. Dedicated guideline repositories over general bibliographic databases were prioritised in order to capture documents explicitly developed to support clinical decision-making rather than primary research studies.

Database	Access	Keywords	Results	Duplicates removed	Records screened
Guideline Central	https://www.guidelinecentral.com/guidelines/	"Birth"	131	0	131
Guidelines International Network	https://guidelines.ebmportal.com	"Birth"	24	0	24
Haute Autorité de Santé - France	https://www.has-sante.fr	"accouchement OR naissance" Filters : recommandations et guides	20	0	20
Joanna Briggs Institute EBP Ovid	https://ovidsp.dc1.ovid.com	"birthing center OR home childbirth OR home birth OR birth center OR out-of-hospital delivery"	21	1	**20**
Trip medical database	https://www.tripdatabase.com	"birthing center" OR "home childbirth" OR "home birth" OR "birth center" OR "out-of-hospital delivery" Filters : Guidelines	39	**1**	**38**

Manual search			27	**11**	**16**

Eligibility criteria

Title and abstract screening focused on identifying documents that explicitly addressed out-of-hospital or home birth, represented formal sets of international guidelines, were published in English, French, Italian, German or Dutch, and provided evidence to support their methodological validity.

During full-text assessment, guidelines were excluded if they were not directly relevant to home birth, lacked supporting evidence, were position statements rather than clinical guidelines, focused exclusively on low-risk definitions without eligibility assessment, addressed system-level organisation rather than individual decision-making, or did not relate to antenatal risk factors.

Screening and full-text assessment were conducted using the Covidence platform (Veritas Health Innovation, Melbourne, Australia). During title and abstract screening, two reviewers independently assessed each record. Discrepancies were resolved through discussion, with involvement of a third reviewer when necessary. For full-text review, one experienced clinical midwife specialised in home birth and one academic midwife independently assessed each guideline.
